# C*ordia lutea* L. Flowers: A Promising Medicinal Plant as Chemopreventive in Induced Prostate Carcinogenesis in Rats

**DOI:** 10.1155/2020/5062942

**Published:** 2020-05-26

**Authors:** Juan Pedro Rojas-Armas, Jorge Luis Arroyo-Acevedo, José Manuel Ortiz-Sánchez, Miriam Palomino-Pacheco, Oscar Herrera-Calderón, James Calva, Agustín Rojas-Armas, Hugo Jesús Justil-Guerrero, Américo Castro-Luna, Julio Hilario-Vargas

**Affiliations:** ^1^Department of Dynamic Sciences, Laboratory of Pharmacology, Faculty of Medicine, Universidad Nacional Mayor de San Marcos, Lima, Peru; ^2^Laboratory of Physiology, Faculty of Medicine, Universidad Nacional Mayor de San Marcos, Lima, Peru; ^3^Laboratory of Biochemistry, Faculty of Medicine, Universidad Nacional Mayor de San Marcos, Lima, Peru; ^4^Faculty of Pharmacy and Biochemistry, Universidad Nacional Mayor de San Marcos, Lima, Peru; ^5^Department of Chemistry and Exact Sciences, Technical Particular University of Loja, Loja, Ecuador; ^6^Instituto Regional de Enfermedades Neoplásicas, Trujillo, Peru; ^7^Department of Physiology, Faculty of Medicine, Universidad Nacional de Trujillo, Trujillo, Peru

## Abstract

The objective of this study was to evaluate the chemopreventive effect of the ethanolic extract of *Cordia lutea* flowers (EECL) on N-methyl-N-nitrosourea- (MNU), cyproterone-, and testosterone-induced prostate cancer in rats. 40 Holtzman male rats were used and assigned to 5 groups (*n* = 8). In Group I, rats received normal saline (10 mL/Kg); Group II: rats were induced for prostate cancer with cyproterone, testosterone, and NMU; Groups III, IV, and V: rats received EECL daily, at doses of 50, 250, and 500 mg/kg body weight, respectively. After the period of treatment, animals were sacrificed by an overdose of pentobarbital and blood samples were collected for determination of prostate-specific antigen (PSA). The prostate was dissected and weighed accurately. The ventral lobe of the prostate was processed for histopathology analysis. The somatic prostate index decreased with EECL at dependent dose, from 0.34 ± 0.04 to 0.23 ± 0.05 (*P* < 0.05). The PSA levels also decreased significantly at doses of 250 and 500 mg/kg. Histopathological analysis showed a decrease in the number of prostatic layers with high-grade prostatic intraepithelial neoplasia (HG-PIN) and low-grade prostatic intraepithelial neoplasia (LG-PIN) at the dose of 500 mg/kg. The ethanolic extract of *Cordia lutea* flowers had a chemopreventive effect on induced prostate cancer in rats.

## 1. Introduction

Cancer is a large group of diseases that can start in almost any organ or tissue in the body when abnormal cells grow uncontrollably, go beyond their usual limits to invade adjacent parts of the body, and/or spread to other organs, the latter. This process is called metastasis and is a major cause of cancer death [1]. Population growth and increased longevity are globally increasing the number of older people; aging is associated with noncommunicable diseases (NCDs), which are responsible for seven out of every 10 deaths in the world [2]. One of the main causes of death from NCDs is cancer, which in 2018 affected 18.1 million people worldwide and produced 9.6 million deaths. Estimating by 2040, these numbers will be almost double, the largest increase being in low- and middle-income countries, where more than two-thirds of cancers in the world will occur [3]. Lung, prostate, colorectal, stomach, and liver cancer are the most common types of cancer in men, while breast, colorectal, lung, cervical, and thyroid cancer are the most common in women [1].

In the United States, cancer is the second leading cause of death, with 1,806,590 new cancer cases and 606,520 cancer deaths projected in 2020, of which 191,930 will be new cases of prostate cancer causing 33,330 deaths [4]. In Peru, death rates from prostate cancer increased from 20.9 (2005–2009) to 24.1 (2010–2014) per 100,000 men, an increase of 15.2%. According to the regions, during the period 2010–2014, the coast had the highest mortality rate (28.9 per 100,000), while the rain forest had the lowest (7.43 per 100,000) [5].

The drugs used in cancer treatment have no selectivity to destroy tumor cells and also damage nontumor cells, producing many side effects, mainly gastrointestinal, neurological, hematological, dermatological, etc. [6,7]. Furthermore, toxicity extends to people who are exposed to the chemotherapy area for job or another reason [8]. On the other hand, the cost of drugs for the treatment of prostate cancer is high [9]. This situation motivates the search for new agents with a better safety profile and lower cost. In this context, an alternative source is natural resources.

*Cordia lutea* (Family: Boraginaceae) is an indigenous plant of Peru, sometimes a shrub or tree, whose flowers are yellow; it is known by the common name of *overo, alkka mallki, biyuyo, caujaro, gomo, yellow gomo, muercielago*, or *ubito*. In traditional Peruvian medicine, *Cordia lutea* has been widely used for the treatment of hepatic disorders [10] and in northern Peru for prostate inflammation [11]. In traditional Peruvian medicine, *Cordia lutea* is known as “*overo*” flower, which have been widely used for the treatment of liver disorders and in northern Peru for prostate inflammation [11].

The phytochemical study of the flower revealed the presence of terpenic and phenolic compounds, such as flavonoids and leucoanthocyanidins, of which the majority compound was rutin [12]. Other research also reported the presence of rutin and quercetin in the whole plant as the main constituents [10]. The anticancer effect of quercetin and rutin has been reported in several animal models, retarding, inhibiting, or suppressing tumor growth [13]. This background motivated us to propose the evaluation of the chemopreventive effect of the ethanolic extract from *Cordia lutea* flowers on NMU-induced prostate cancer in rats.

## 2. Material and Methods

### 2.1. Preparation of Extract of *Cordia lutea* Flowers

*Cordia lutea* L. *(C. lutea)* flowers were collected in the city of Trujillo, Peru. The material was identified at the Herbarium of the National University of San Marcos, Lima, Peru. The flowers were washed, dried at 40°C, and pulverized in an electric mill. Phytochemicals were extracted in 96% ethanol. The extract was collected, filtered, and concentrated on a rotary evaporator; finally, it was stored in aliquots and kept in freezer until further use.

### 2.2. Phytochemical Analysis of Ethanol of Extract *Cordia lutea*

The chemical components of the ethanol extract were analyzed on a gas chromatograph (Agilent Technologies 6890N), coupled to a mass spectrometer 5973N (Santa Clara, CA, USA) and equipped with a DB-5MS capillary column (5% phenyl methyl silox, 30 m, 0.25 mm internal diameter, 0.25 *μ*m film thickness; J&W Scientific, Folsom, CA, USA). For the separation of volatile components, the following temperature program was used: 5 min at 60°C, 3°C/min up to 165°C, and 15°C/min up to 250°C and kept for 10 min. Injector and detector temperatures were maintained at 220°C. The carrier gas was helium, at a flow rate of 1 mL/min. The injector was operated in split mode, with a division ratio of 1 : 50. The acquisition mass range was set at 40–350 m/*z*. Ionization mode: impact of electrons (70 eV). The extract was diluted to 1 : 100 v/v in dichloromethane (Fisher Scientific, 99.9% pure) and 1 *µ*L of the solution was injected [14].

### 2.3. Animals

Healthy male Holtzman adult rats weighing 200 ± 20 g were used. The animals were purchased from the National Institute of Health, housed in polypropylene cages and air-conditioned environment with a 12 h light/dark cycle. They were allowed free access to drinking water throughout the experimental period. The animals were fed with standard feed for rats. The specifications and recommendations proposed by the guide for the Care and Use of Laboratory Animals were followed and in compliance with the current regulations of the Animal Protection Law (Law no. 27265) [15].

### 2.4. Induction of Prostate Cancer

Prostate cancer induction was carried out with carcinogen and hormone, in a sequential process that began with temporary chemical castration with cyproterone acetate, followed by prostate stimulation with testosterone propionate, and finally carcinogenesis with N-methyl-N-nitrosourea (MNU). The method of Sharmila et al. [16] and Banudevi et al. [17] was followed with slight modification; rats received cyproterone acetate (50 mg/kg body weight in sesame oil) daily by intraperitoneal injection for 18 consecutive days; one day after the final dose of cyproterone acetate, rats received daily subcutaneous injections of testosterone propionate (100 mg/kg of weight in sesame oil) for 3 days; the day after testosterone propionate administration, each rat received a single intraperitoneal injection of N-methyl-N-nitrosourea (MNU) at a dose of 50 mg/kg body weight in sterile saline, pH 5.0. The cancer developed in the following 5 months and was evidenced at the end of this period through the histopathological study.

### 2.5. Experimental Design

A total of 40 rats were randomly assigned to 5 groups (*n* = 8). Group I: rats received NS (normal saline); Group II : rats were induced for prostate cancer with cyproterone, testosterone, and NMU; Groups III, IV, and V: rats were induced for cancer and received treatment with the flower extract of *C. lutea* daily in doses of 50, 250, and 500 mg/kg of body weight, respectively, starting after the induction of cancer and it was continued for 5 months. After the treatment period, rats were sacrificed by pentobarbital overdose and blood samples were collected for the serum determination of PSA and biochemical and hematological parameters. The prostate was dissected from the adherent connective tissue, washed several times with physiological solution, weighed exactly, and separated. The ventral lobe of the prostate was processed for histopathological examination.

### 2.6. Biochemical and Hematological Tests

Blood collection in rats was performed by intracardiac puncture; the animals were previously subjected to a state of general anesthesia (Ether chambers). Hematological tests were performed on a KT-6400 Automatic Hematology Analyzer (Genius®, Med Equipment) and the biochemical tests were performed on an EMP-168 Model Semi-Automatic Biochemical Analyzer (Ivdiagnostik®, Emperor Medical) according to the manufacturer's specifications.

Serum prostate-specific antigen (PSA) levels were quantified using the Chemiluminescence Immunoassay (CLIA) System, using the Maglumi 1000 (Snibe Diagnostic®) equipment, following the manufacturer's specifications.

### 2.7. Histopathology Study

The fixed ventral prostate lobe was sequentially dissected and the samples embedded in paraffin and sectioned at 4 *μ*m were placed in the sheets and colored with Hematoxylin and Eosin. The histopathological study was performed by optical microscopy.

### 2.8. Statistical Analysis

Data were presented as mean ± standard deviation. They were analyzed by one-way analysis of variance (ANOVA). The statistical significance between the means was determined by a post hoc Tukey test. The statistical software SPSS version 19 was used. Values of *P* < 0.05 were considered statistically significant.

## 3. Results

### 3.1. Phytochemical Analysis of Ethanol Extract of *Cordia lutea* by GC/MS

The ethanolic extract of the *Cordia lutea* flower had a yield of 13.55%. The spectra of the unknown components were compared to the spectrum of the known components stored in the NIST library. A total of four natural compounds were identified from flower's ethanol extract (Table 1). Retention Time (RT) for polar components varied between 29.99 min and 36.03 min, including the major compounds gibberellic acid (31.67%; RT: 15.75) and hexamethylcyclotrisiloxane (30.88%; RT:36.03). The major compounds mentioned below and others with their RT, molecular formula (MF), and molecular weight (MW) were shown in Table 1.

### 3.2. Biochemical and Hematological Analysis

Table 2 showed that there was not significant decrease (*P* > 0.05) in RBC, WBC, Hemoglobin, Hematocrit, Neutrophils, Eosinophils, Basophils, Monocytes, Lymphocytes, and Platelets of the animals treated with *Cordia lutea* (50, 250, and 500 mg/Kg) when compared to the control group.

In Table 3, the result of serum liver and kidney function parameters of rats administered extract of *Cordia lutea* showed a significant decrease (*P* < 0.05) in aspartate aminotransferase (AST), alanine aminotransferase (ALT), and alkaline phosphatase (ALP) compared to the control animals. All other parameters tested were not significantly different in all the groups compared to control.

At all the doses tested, there were significant reductions (*P* < 0.05) in the triglycerides and LDL level of the rats compared to the control (Table 3). Animals in the 250 and 500 mg/kg groups witnessed significant increase (*P* < 0.05) in HDL-C concentration compared to the control group.

### 3.3. Morphological and Histopathology Study of Animals

**Figure 1:**
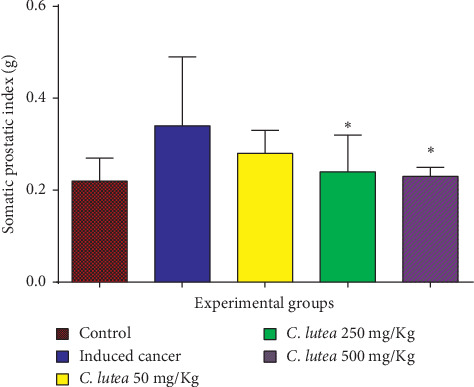
Somatic prostate index in rats with induced prostate cancer after 5 months of treatment with *Cordia lutea*. ^*∗*^(*P* < 0.05) vs. induced cancer group.

**Figure 2:**
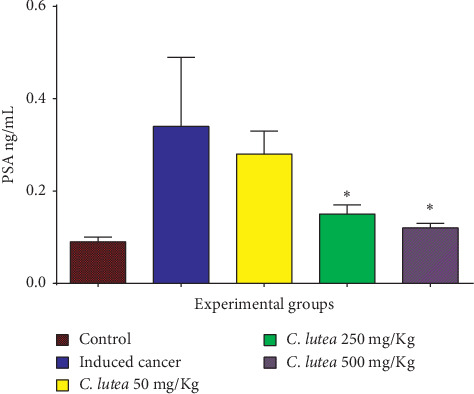
PSA levels in rats with induced prostate cancer after 5 months of treatment with *Cordia lutea*. ^*∗*^(*P* < 0.05) vs. induced cancer group.

The somatic prostate index (prostate volume/body weight *×* 100) decreased with the treatment of *Cordia lutea* in a dose-dependent manner, being significant with the dose of 250 and 500 mg/kg. The best effect occurred with the dose of 500 mg/kg, where a decrease was observed from 0.34 ± 0.04 to 0.23 ± 0.05 (*P* < 0.05) (Figure 1). Serum PSA levels decreased significantly with the dose of 250 and 500 mg/kg (Figure 2).

**Figure 3:**
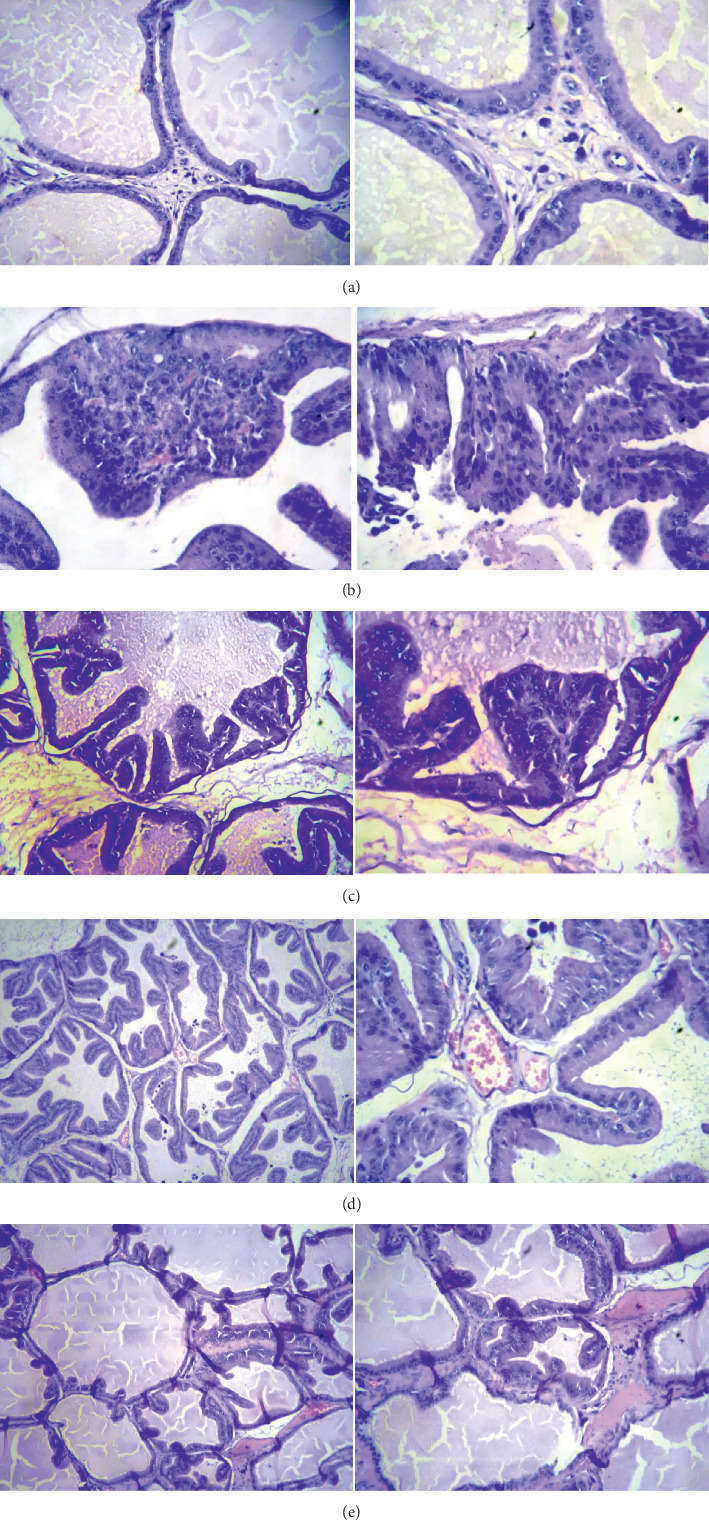
Microphotography of rat's prostate stained with Hematoxylin and Eosin (H&E), at 10 X and 40 X magnification. (a) Physiological saline 2 mL/kg. Normal. Cubic cells, not columnar. (b) Prostate tissue with induced cancer. Many layers are observed, cells in mitosis; large number of cells with high-grade prostatic intraepithelial neoplasia (HG-PIN) and low-grade prostatic intraepithelial neoplasia (LG-PIN). (c) *Cordia lutea* 50 mg/kg, intraepithelial neoplasia of low and somewhat grade is observed. (d) *Cordia lutea* 250 mg/kg, basically hyperplasia is observed, high- and low-grade foci are not reached. (e) Induced + *Cordia lutea* 500 mg/kg, hyperplasia is observed; it remains in hyperplasia; it does not reach high-grade or low-grade foci.

Histopathological analysis will show a decrease in the number of layers, as well as high-grade prostatic intraepithelial neoplasia HG-PIN and low-grade prostatic intraepithelial neoplasia (LG-PIN). It was considered the best effect according to the evaluated doses (Table 4 and Figure 3).

## 4. Discussion

In the present investigation, the presence of gibberellic acid was found as chemical component, a compound that has not been reported by other researchers who rather found the presence of terpenic and phenolic compounds, such as flavonoids and leucoanthocyanidins, of which the majority compound was rutin [12].

Other research also reported the presence of rutin and quercetin in the whole plant as the main constituents [10]. This situation could be due to the method used in the investigation, since in our case the gas chromatography technique coupled to mass spectrometry (GC/MS) was used, while the presence of rutin and quercetin was determined by UPLC/MS.

The cancer-inducing model used in this study was able to produce an intraepithelial neoplasm, an infiltrating cancer that is divided into high grade and low grade; foci developed, reaching areas with hyperplasia and areas where there was a noticeable change with more than five layers. Treatment with *Cordia lutea* extract showed a favorable effect on the neoplastic process, evidenced by the reduction in the number of layers and high-grade prostatic intraepithelial neoplasia (HG-PIN), as well as low-grade prostatic intraepithelial neoplasia (LG-PIN) (Table 2 and Figure 3). Moreover, serum levels of PSA and somatic prostate index (prostate volume/body weight of the rat) decreased significantly the effect of treatment with *Cordia lutea* (Figures 1 and 2). These results relate to the observed histopathologic changes.

It has been reported that a large number of phytochemicals have anticancer properties, including polyphenols due to their free radical sequestration activity that confers antioxidant activity [18]. It is important to highlight the role of reactive oxygen species (ROS) in the activation of NF-kB and the subsequent transcription of more than 200 genes that suppress apoptosis and induce cell transformation, proliferation, invasion, metastasis, chemoresistance, radioresistance, and inflammation [19]. It has been shown that polyphenols can inhibit the growth of cancer cells by interacting with multiple signaling pathways, including those of NF-kB [20].

Various investigations have shown the anticancer effect of routine flavonoids and quercetin in several neoplasms. Rutin induced apoptosis in HT-29 human colon cancer cells, mediated by the receptor and mitochondria-mediated apoptotic pathways [21]. Likewise, it produced an antineuroblastoma effect via the arrest of the cell cycle progression in the G2/*M* phase and also induced cell apoptosis as well as the regulation of apoptosis-related genes [22]. Rutin, via nonselective inhibition of P-glycoprotein (P-gp) and breast cancer resistance protein pump (BCRP), efficiently reverses multidrug resistance and restores chemosensitivity to cyclophosphamide and successfully stops cell cycle progression [23].

It also significantly inhibited the progression of human hepatocellular carcinoma HEPG2 cells, was remarkably effective on migration, colony formation, and invasive potential of HEPG2 cells, and increased apoptosis. In addition, rutin was found to be a potent CYP3A4 inhibitor and activator of CYP1A1 and of antioxidant enzymes glutathione S-transferases (GSTs) and NADPH quinone oxidoreductase I (NQO1) [24]. Furthermore, high-dose quercetin reduced colorectal carcinogenesis in rats; it is noteworthy to mention that this flavonoid has many intracellular targets in the treatment of cancer including proteins involved in apoptosis, cell cycle, detoxification, antioxidant, replication, and angiogenesis [25, 26]. The flavonoids quercetin and rutin showed antiangiogenic activity in the chorioallantoic membrane model and antioxidant and anticancer activity [27].

One study has reported that in prostate cancer cells, quercetin can exert its chemopreventive effect by 3 mechanisms: (a) inhibiting the activity of CYP1A1 and CYP1B1 (overexpressed in human cancer cells) and therefore reducing the formation of mutagenic intermediates and carcinogens of polycyclic aromatic hydrocarbons (PAHs), heterocyclic amines, and estradiol; (b) regulating the high peroxiredoxin 3 (Prx III), thereby reducing intracellular H_2_O_2_ levels leading to cell proliferation inhibition; and (c) counteracting the effects mediated by the ubiquitous environmental contaminant benzo[a]pyrene (BaP), whose exposure is associated with prostate carcinogenesis, on Prx I and Prx II peroxiredoxins or interacting directly with ROS mediated by BaP and preventing oxidative damage [28].

## 5. Conclusion

In conclusion, under the experimental conditions the ethanolic extract of *Cordia lutea* has chemopreventive effect on NMU-induced prostate cancer in rats. It is likely that flavonoids quercetin and rutin in the extract of *Cordia lutea*, for its anti-inflammatory, antiangiogenic, apoptotic, and antioxidant properties, are partly responsible for the antineoplastic effect observed in this study.

## Figures and Tables

**Table 1:** Major components detected in the ethanol extract of *Cordia lutea*.

Name of compound	Retention time	Area (%)	Molecular formula	Molecular weight
Hexadecanoic acid, butyl ester	29.99	5.89	C_20_H_40_O_2_	118.17
Butyl 9,12,15-octadecatrienoate	31.71	4.29	C_22_H_38_O_2_	312.53
Gibberellic acid	31.67	15.75	C_19_H_22_O_6_	346.40
Hexamethylcyclotrisiloxane	36.03	30.88	C_6_H_18_O_3_Si_3_	315.25

^a^RT: retention time determined relative to a DB-5MS (5% phenyl methyl silox). ^b^Identification method: MS, comparison of mass spectra with those listed in the NIST11 and Wiley 9 libraries.

**Table 2:** Hematological parameters of rats induced with prostate cancer after treatment with *Cordia lutea* for 5 months.

Parameters	Control (NS)	Induced cancer	*C. lutea* 50 mg/kg	*C. lutea* 250 mg/kg	*C. lutea* 500 mg/kg
RBC (x106/ul)	6.13 ± 0.79	6.32 ± 0.17	6.12 ± 0.34	6.28 ± 0.11	6.24 ± 0.13
WBC (x103/ul)	7.24 ± 0.51	6.98 ± 0.53	7.17 ± 0.30	6.68 ± 0.27	6.72 ± 0.43
Hemoglobin (g/dL)	14.97 ± 0.75	15.15 ± 0.23	14.67 ± 0.32	15.13 ± 0.31	14.80 ± 0.52
Hematocrit (%)	46.67 ± 2.31	48.33 ± 1.53	45.67 ± 2.52	47.67 ± 1.53	46.00 ± 1.73
Neutrophils (%)	24.67 ± 3.21	24.33 ± 3.51	24.00 ± 3.61	26.33 ± 4.93	27.33 ± 4.16
Eosinophils (%)	3.67 ± 0.58	4.00 ± 1.00	3.67 ± 1.15	3.33 ± 1.53	3.67 ± 1.53
Basophils (%)	1.33 ± 0.58	1.67 ± 0.58	1.67 ± 0.58	1.67 ± 0.58	1.33 ± 0.58
Monocytes (%)	4.33 ± 1.53	5.00 ± 1.00	4.00 ± 1.00	4.33 ± 0.58	4.67 ± 1.15
Lymphocytes (%)	66.00 ± 3.00	65.00 ± 4.36	66.67 ± 5.51	64.33 ± 3.79	63.00 ± 4.58
Platelets (×10^3^/ul)	534.00 ± 24.25	551.33 ± 20.55	560.00 ± 20.00	542.00 ± 25.24	541.00 ± 11.53

Values are expressed as mean ± SD, no significance when compared with control. RBC: red blood cell; WBC: white blood cell.

**Table 3:** Biochemical parameters of experimental groups treated with *Cordia lutea* for 5 months.

Parameters	Control (NS)	Induced cancer	*C. lutea* 50 mg/kg	*C. lutea* 250 mg/kg	*C. lutea* 500 mg/kg
AST (IU/L)	164.19 ± 8.31	199.01 ± 7.76	184.43 ± 12.10	175.40 ± 7.55^*∗*^	173.50 ± 5.07^*∗*^
ALT (IU/L)	66.63 ± 3.46	88.57 ± 10.77	85.63 ± 7.40	81.10 ± 3.29	78.80 ± 12.39^*∗*^
Alkaline phosphatase (IU/L)	311.2 ± 11.15	330.73 ± 5.93	320.60 ± 17.17	318.67 ± 16.04^*∗*^	316.43 ± 9.68^*∗*^
Total bilirubin (mg/dL)	0.71 ± 0.07	0.79 ± 0.09	0.75 ± 0.05	0.73 ± 0.09	0.73 ± 0.05
Total protein (g/dL)	6.80 ± 0.14	7.18 ± 0.22	7.11 ± 0.24	7.08 ± 0.12	6.98 ± 0.19
Albumin (g/dL)	3.96 ± 0.12	4.09 ± 0.07	3.90 ± 0.06	3.88 ± 0.05	3.93 ± 0.11
Cholesterol (mg/dL)	74.33 ± 7.02	89.33 ± 7.77	88.87 ± 11.20	87.73 ± 9.40	85.80 ± 3.86
Triglycerides (mg/dL)	107.00 ± 7.94	133.33 ± 6.66	129.67 ± 5.51	127.67 ± 7.02	120.87 ± 8.95^*∗*^
HDL (mg/dL)	31.00 ± 1.73	29.00 ± 2.00	30.83 ± 3.68	30.67 ± 1.15	32.67 ± 2.08^*∗*^
LDL (mg/dL)	21.93 ± 7.31	33.67 ± 8.42	32.10 ± 12.18	31.53 ± 9.79	28.96 ± 6.94^*∗*^
Glucose (mg/dL)	99.67 ± 2.52	97.00 ± 5.29	101.33 ± 2.52	104.33 ± 7.37	100.67 ± 2.31
Urea (mg/dL)	35.77 ± 3.13	39.13 ± 2.68	38.87 ± 4.75	41.80 ± 4.61	38.17 ± 2.46
Creatinine (mg/dL)	0.77 ± 0.04	0.84 ± 0.06	0.83 ± 0.06	0.84 ± 0.05	0.80 ± 0.04^*∗*^

Values are mean ± SD (expressed as mean ± standard deviation), significance when compared with control ^*∗*^(*P* < 0.05). One-way ANOVA followed by Tukey's test. NS: normal saline; AST: aspartate aminotransferase; ALT: alanine aminotransferase; HDL: high-density lipoprotein; and LDL: low-density lipoprotein.

**Table 4:** Histopathological analysis of prostate tissue in rats with prostate cancer and treated with *Cordia lutea* for 5 months.

Group	Lumen	Epithelium	Layers	LG-PIN	HG-PIN
NS	Regular	Cubic	1	Negative	Negative
Induced cancer	Papillary	Columnar	>5	Positive ++/+++	Positive ++/+++
*Cordia lutea* 50 mg/kg	Papillary	Columnar	2–3	Positive ++/+++	Positive +/+++
*Cordia lutea* 250 mg/kg	Papillary	Columnar	2–3	Positive +/+++	Negative
*Cordia lutea* 500 mg/kg	Papillary	Columnar	1–2	Negative	Negative

NS = normal saline. HG-PIN = high-grade prostatic intraepithelial neoplasia. LG-PIN = low-grade prostatic intraepithelial neoplasia.

## Data Availability

All data used to support the findings of this study can be made available from the corresponding author upon request (oherreraca@unmsm.edu.pe).

## Abbreviations

NMU: N-Methyl-N-nitrosourea
EECL: Ethanolic extract of *Cordia lutea*
NF-kB: Nuclear factor kappa-light-chain-enhancer of activated B cells
ANOVA: Analysis of variance
NS: Normal saline
HG-PIN: High-grade prostatic intraepithelial neoplasia
LG-PIN: Low-grade prostatic intraepithelial neoplasia.
